# Mechanisms of Degradation of Insoluble Dietary Fiber from Coconut Chips by Ultra-High Pressure

**DOI:** 10.3390/foods13193174

**Published:** 2024-10-06

**Authors:** Qiaozhu Wei, Jingtao Cui, Weimin Zhang, Lianzhou Jiang, Tian Li

**Affiliations:** 1 Key Laboratory of Food Nutrition and Functional Food of Hainan Province, School of Food Science and Engineering, Hainan University, Haikou 570228, China; 16635711513@163.com (Q.W.); jingtaocui@126.com (J.C.); zhwm1979@163.com (W.Z.); 2 College of Food Science, Northeast Agricultural University, Harbin 150030, China; jlzname@163.com

**Keywords:** ultra-high pressure, coconut chips, insoluble dietary fibers

## Abstract

Coconut chips are a popular leisure food, but the residual crumbly feeling after chewing affects the eating experience. To address this problem, we investigated the mechanism of degradation of insoluble dietary fiber (IDF) from coconut chips by ultra-high pressure (UHP). The optimal conditions for UHP treatment were 100 MPa and 40 min. After UHP treatment, the hardness decreased by 60%, and the content of soluble dietary fiber (SDF) increased by 55%. So far, the meaning of SDF has not been defined. The microstructure of IDF was damaged and the surface was rough. There was no obvious change in the chemical structure. The position of the characteristic diffraction peaks was basically unchanged, but the crystallinity dropped by almost three times. The thermal stability decreased, and the composition of the monosaccharides changed. Together, UHP treatment can improve the problem of the residual crumbly feeling after chewing coconut chips and improve the quality of the product.

## 1. Introduction

The coconut tree is a perennial evergreen tree in the palm family, and the coconut is the fruit of the coconut tree [1]. It is a major woody oil crop and is widely used in the food industry. Among them, coconut chips are a thin, crunchy, and flavorful snack food that is popular among people. However, coconut chips have a crumbly feeling after chewing, which seriously affects their consumption experience. A study has revealed that dried coconuts contain lipids, proteins, and high levels of dietary fiber [2]. The research explains that insoluble dietary fiber (IDF) is responsible for the rough texture. Therefore, there is an urgent need to address this issue by degrading the IDF.

In 2009, the Codex Alimentarius Commission (CAC) defined dietary fiber as a carbohydrate polymer with ten or more monomeric units that is not hydrolyzed by endogenous enzymes in the human small intestine [3]. Base on the solubility of dietary fiber, it can be classified into two main categories: soluble dietary fiber and insoluble dietary fiber. Soluble dietary fiber mainly includes pectin, gum, mucilage, fructans, and some resistant starch [4]. Sugar chains in insoluble dietary fiber are bound to each other through dense hydrogen bonds, forming a hydrophobic crystalline structure, which can resist hydrolysis by exogenous glucosidases. Insoluble dietary fibers mainly include cellulose, hemicellulose, and lignin [5]. Currently, the major chemical, biological, and physical methods have been reported to degrade IDF [6]. The chemical methods include acid and alkali methods, carboxymethylation (CM), hydroxymethylation (HM), cross-linking (CL), and so on [7]. The available reports have shown a 56.5% decrease in IDF content in wheat and 71.17% in sorghum after acid-base treatment [8]. The use of chemical methods such as CM and HM similarly reduces the IDF content and improves the physicochemical properties of oat dietary fiber [9]. However, the chemical modification process may lead to reagent residues and environmental pollution, which is unsafe and affects the flavor of food [10]. The primary biological method is enzymatic. In the present research, 15.65% and 12.26% of IDF was degraded in carrot (Cellulase (90 U/g) and xylanase (48 U/g)) [11] and wakame (xylanase (20 U/g) and Cellulase (40 U/g)) [12], respectively, by the combined enzymatic method. However, the enzyme method is expensive and time-consuming, which is not conducive to large-scale production [13]. The physical methods include steam blasting, extrusion treatment, ultrasonic methods, etc. The steam blasting treatment resulted in 8.22–25.94% degradation of IDF in rice bran [14]. Moreover, steam blasting and extrusion degraded 6% and 12% of IDF in coconuts, respectively [15]. The ultrasound treatment degraded 7% and 5.13% of IDF in prickly pear pomace [16] and corn husk [17]. In the existing report, the ultra-high pressure (UHP) treatment was reported to degrade 36.44% of IDF in purple potato [18]. The physical method of UHP degradation of IDF is superior to other methods. In comparison with chemical and biological methods, physical methods have the advantages of simplicity of operation, low cost, and sustainability [19].

Based on the above reasons, coconut chips were used as an experimental raw material to be modified by UHP treatment in this study. In order to achieve the purpose of improving the crumb sensation of coconut chips after chewing, the coconut chips were subjected to sensory evaluation, determination of major components (moisture, fat, protein), texture determination, and determination of dietary fiber content. The IDF microstructure, infrared spectral properties, crystalline properties, thermal stability, and monosaccharide composition of coconut chips were then analyzed separately to determine the optimal UHP treatment conditions. Meanwhile, the mechanism of reducing the brittleness of coconut chips was elucidated.

## 2. Materials and Methods

### 2.1. Materials

Raw coconut meat (7–8 mature) was purchased from Hainan Nanguo Co., Ltd. (Haikou, China). 1-phenyl-3-methyl-5-pyrazolinone (PMP) and trifluoroacetic acid (TFA) were purchased from Sigma Reagents (Livonia, MO, USA). Sodium hydroxide, hydrochloric acid, disodium hydrogen phosphate, sodium dihydrogen phosphate, petroleum ether, and ethanol were purchased from Chemical Reagent Factory (Guangzhou, China). All the above reagents were of analytical grade. Other reagents were purchased from Aladdin Co., Ltd. (Shanghai, China).

### 2.2. UHP Pre-Treatment and Preparation with Coconut Chips

The preparation of coconut chips consists of three processes: UHP pre-treatment, sugar boiling, and roasting. The coconut chips (30 g) were weighed and vacuum-molded into 10 cm × 6 cm bags. The samples were then mixed with distilled water and placed in ultrahigh-pressure equipment at 100 MPa for different periods of time (0, 20, 40, and 60 min). At the end of the UHP treatment, the water was poured off, and the samples were percolated in a 25% sugar solution for 20 min. The samples were then transferred to tin foil and dried in an electrically heated oven at 75 ± 1 °C for 45 min. Finally, the samples were baked in an oven at 150 ± 1 °C for 10 min. After cooling, the samples were placed in a sealed bag and stored in a dry and ventilated environment.

### 2.3. Color

The samples were determined using a corrected hand-held colorimeter. The color values were detected according to the CIE Lab color space of L*, a*, and b*, where L*, a*, and b* were represented as luminance (darkness (0) to brightness (100)), green to red (greenness (−a*) to redness (+a*)), and blue to yellow (blueness (−b*)) to yellowish (+b*)), respectively. Δ*E* represented the difference in color between individual samples, which was calculated using the formula reported by Sara E. Yeager et al. [20]:

(1) $$∆E=\sqrt{(L^{*}-L_{0}^{*}{)}^{2}+{\left(a^{*}-a_{0}^{*}\right)}^{2}+{\left(b^{*}-b_{0}^{*}\right)}^{2}}$$

where *L*_0_*, *a*_0_*, and *b*_0_* represent the color of coconut chips in the untreated group.

### 2.4. Hardness

The hardness of coconut chips was measured using a texture analyzer (TA.XT Plus, Stable Micro Systems Ltd., Godalming, UK). The target type and target value were displacement and 10.0 mm, respectively. The pre-test speed, intra-test speed, and post-test speed were 1.0 mm/s, 0.5 mm/s, and 1.0 mm/s, respectively. Hardness was expressed as the maximum force required for the first compression.

### 2.5. Determination of Major Components

#### 2.5.1. Moisture

The samples were broken into a powder and passed through an 80-mesh sieve. The powder samples were weighed in a clean glass dish. The cap was placed diagonally against the side of the flask in an electrically heated thermostatic drying oven. The temperature was set to 105 ± 1 °C. After drying for 4 h, the cap was removed and placed in a desiccator to cool for 0.5 h. Then, the samples were removed and weighed again. The above procedures were repeated until the weight was constant.

#### 2.5.2. Fat

Powder samples (3 g) were weighed and wrapped in filter paper. Filter paper packs were dried in an electric oven (105 ± 1 °C) for 2 h before being placed in extraction tubes. Petroleum ether (120 mL) was added to the test tube and extracted for 16 h at 60 ± 1 °C in a water bath. After extracting, the petroleum ether was removed from the flask using a rotary evaporator (40 ± 1 °C). The residual solvent was removed from the water bath, and the flask was dried in an electrically heated oven at 105 ± 1 °C for 2 h. The samples were cooled in a desiccator and weighed. This operation was repeated until the weight was constant. The difference in mass before and after the flask was taken as the fat content (g/100 g).

#### 2.5.3. Protein

The protein was determined using the Dumas combustion method [21]. The samples after oil removal and dehydration were wrapped in nitrogen-free tin foil, which was compacted in a pressurized sampler. The instrument was calibrated using aspartic acid as the standard, and the reactor was set at 1200 ± 1 °C with a flow rate of O_2_ (400 mL/min). Helium was used as the delivery gas, and samples were detected through a thermal conductivity detector.

### 2.6. The Extraction of Insoluble and Soluble Dietary Fiber

DF included insoluble dietary fiber (IDF) and soluble dietary fiber (SDF). The samples were weighed into a beaker and degreased by n-hexane in proportion of 25 mL/g for 24 h, then removed and dried naturally. The DF was extracted by a modified method according to Hemanta Chutia et al. [22]. The degreased samples were added to a 6% *w*/*v* NaOH solution at a ratio of 1: 10 and soaked for 2 h at 70 ± 1 °C. Then, the samples (60 ± 1 °C) were prepared using ultrasound-assisted alkaline extraction under an amplitude of 40% for 35 min. After filtration, the precipitate was washed continuously with distilled water at least 5–7 times until it was near neutrality, and then the samples were dried. The SDF of the filtrate was precipitated with four times the volume of ethanol, and the mixture was allowed to stand overnight before it was filtered and dried.

### 2.7. Structural Characterisation

#### 2.7.1. Scanning Electron Microscopy (SEM)

The microscopic morphology of IDF was observed by SEM (Sirion 200, FEI Corp., Hillsboro, OR, USA). The dried and grinded samples were fixed on a specimen holder secured with double-sided conductive tape. The specimen holder was covered with an ion-sputtered gold plating layer. The micrographs of the IDF samples were taken under accelerating voltage conditions of 10.0 kV at 200 and 350 magnifications.

#### 2.7.2. Fourier-Transform Infrared Spectroscopy (FTIR)

The dried sample (2 mg) was weighed and mixed with dried KBr (200 mg) and then milled until homogeneously pressed into thin tablets (1–2 mm). The spectra were obtained in the scanning wave number range of 4000–400 cm^−1^ with a resolution of 16 cm^−1^ [23].

#### 2.7.3. X-ray Diffraction (XRD)

The crystal structure of the IDF samples was characterized based on the method of Zhang et al. [24]. with slight modifications. The ground samples were added into the sample tanks, and the samples’ surfaces were flattened by a smooth glass plate. The sample plates were inserted into the test base of the instrument. The test conditions were as follows: the radiation source was monochromatic Cu-Kα with a theta-compensated slit at a wavelength of 0.1542 nm, a current of 30 mA, and a voltage of 30 kV. The diffraction angle (2θ) ranged from 10–60 °, and the scanning speed was 5°/min. The crystallinity index was calculated using the Hermans–Weidinger method.

### 2.8. Thermal Gravimetry (TG)

The thermodynamic stability of the IDF samples was characterized with the use of thermogravimetric (TG) analysis, where high-purity nitrogen was used as a purge gas to exclude air prior to the experiment. The sample of IDF (10 mg) was weighed and heated from 50 °C to 700 °C in a nitrogen atmosphere at a rate of 10 °C/min [25].

### 2.9. Differential Scanning Calorimetry (DSC)

The thermal stability of the samples was analyzed using a DSC. The IDF (3–10 mg) was weighed and placed in aluminum trays, which was sealed with an aluminum cap. Then, the aluminum trays were pressed. The sealed empty aluminum boxes were placed together in the sample holder as the reference standards. The temperature was increased at a rate of 10 °C/min (100–400 °C), and the nitrogen gas was introduced at a rate of 30 mL/min.

### 2.10. The Monosaccharide Composition of IDF

The monosaccharide composition of IDF was analyzed according to the modified method of Min X et al. [26]. The sample (10 mg) was taken in a hydrolysis tube, and trifluoroacetic acid (2 mol/L, 5 mL) was added. The tube was sealed with N_2_ (10 L/min, 1 min) and hydrolyzed for 2 h at 110 °C. After cooling, the liquid (1 mL) was removed, and methanol (1 mL) was added. The tube was blown dry with N_2_ in a water bath (70 ± 1 °C). The procedures were repeated twice to remove TFA. The residue was fully dissolved by 1 mL of a 0.3 mol/L NaOH solution as the polysaccharide hydrolysate. Reduced monosaccharides were determined by PMP derivatization, and the samples were analyzed using an Agilent 1100 equipped with a C18 column (250 mm, 4.6 mm, 5 μm). The column temperature was 30 °C, and the detection wavelength was 250 nm. The mobile phases of A and B were 100 mM sodium phosphate buffer (pH = 6.6) and acetonitrile, respectively. The flow rate and injection volume were 1 mL/min and 5 μL, respectively. The gradient elution conditions were: 85% A for 10 min, 83% A for 20 min, 80% A for 5 min, 60% A for 1 min, and 85% A for 4 min.

### 2.11. Data Analysis

All the measurements were performed at least in triplicate, and the results were reported as average values with standard deviation. The data were processed using GraphPad Prism 5.0 (San Diego, CA, USA), and the analysis of variance (ANOVA) was conducted using SPSS statistical software (version 21.0, IBM SPSS Inc., Chicago, IL, USA) to test the significance. For this purpose, Duncan’s test with a significance level of 95% (*p* < 0.05) was adopted.

## 3. Results and Discussion

### 3.1. Color

**Figure 1:**
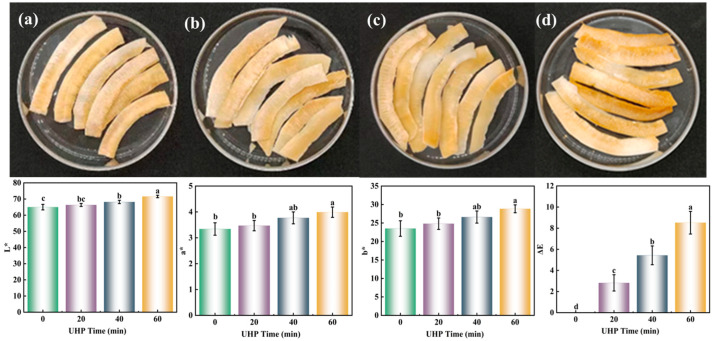
The color of coconut chips under different UHP times. (**a**) Untreated group; (**b**) ultra-high pressure for 20 min; (**c**) ultra-high pressure for 40 min; (**d**) ultra-high pressure for 60 min. The different letters indicate the significance between L* and L*, a* and a*, b* and b*.

The most intuitive way to determine the quality of coconut chips is to observe the appearance of the chips. Color is one of the important attributes of coconut chips. As shown in Figure 1, the coconut crisps after different treatments had a light golden color. The L*, a*, b* of coconut chips after 40 min and 60 min of treatment showed a significant increase (*p* < 0.05) as compared to those of the untreated group. It showed a darker yellowish brown color. This may be due to the UHP effect resulting in cell rupture and easier penetration of the sugar solution. This made the coconut chips more susceptible to caramelization during baking. There was no significant difference in the L*, a*, b* values of coconut chips between the UHP-treated for 20 min and untreated groups (*p* > 0.05), which may be due to the shorter treatment time, which did not cause serious damage to the structure.

### 3.2. Statistical Analysis of the Hardness Results

**Figure 2:**
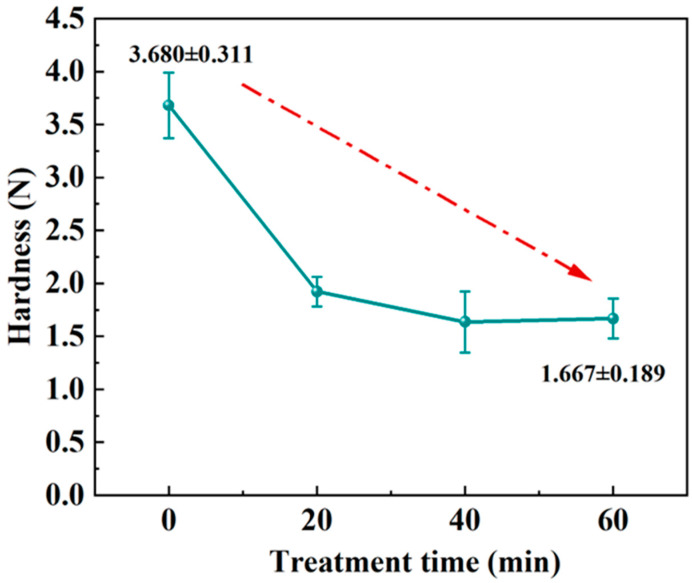
The hardness of coconut chips under different UHP times.

As can be seen in Figure 2, hardness reduced to a certain value and then ceased to change with the increase in treatment time. In this instance, the hardness of all samples from the untreated group was significantly higher (*p* < 0.05) than that of the treated group. There was no significant difference in the hardness (1.637 ± 0.288 ~1.921 ± 0.139) of coconut chips between the three treatment groups (*p* < 0.05). This could be due to the extremely high hydrostatic pressure generated during the UHP process and the accompanying mechanical effects such as friction and impact. This causes the originally compact structure of the coconut chips to become loose, which caused the hardness to drop. There was no difference between the three treatment groups, with the lowest hardness after 40 min of treatment. The hardness of the chips was reduced as a result of the UHP pre-treatment, similar to the findings of Zhang et al. [27]. In order to further explore the intrinsic mechanism of the effect of UHP pre-treatment process on the quality of coconut chips, we carried out further compositional and structural characterization of coconut chips.

### 3.3. Determination of Major Components

The main components in coconut chips were determined after clarifying the effect of UHP pre-treatment on the sensory evaluations and hardness of the coconut chips. From Table 1, the moisture content of the UHP-pre-treated coconut chips was significantly lower than that of the untreated coconut chips (*p* < 0.05). This may be due to the strong mechanical action of the UHP destroying the previously dense structure and the enhanced drying effect [28]. The protein content of coconut chips in the untreated group was 7.57 ± 0.88%, which did not differ significantly (*p* < 0.05) from that of the treated group. According to the work of Kang et al. [29], the effect of UHP treatment mainly targets the secondary and tertiary structures of proteins, which exposes more hydrophobic groups. The fat content of coconut chips in the untreated group was 41.37 ± 0.95%, which was lower than that of the untreated group for 60 min of treatment. This may be due to the prolongation of the UHP treatment time, which resulted in rupture of the coconut chips cells and spillage of fat from their interior.

### 3.4. Dietary Fiber Fraction

In order to further identify the intrinsic mechanism by which UHP affects the quality of coconut chips, the dietary fiber was extracted from coconut chips and structurally characterized. The reasons for the improvement of the texture of coconut chips by UHP pre-treatment were further explained. The extraction rates of IDF, SDF, and TDF in different treatment groups are evident from Table 2. The SDF extraction rates of the samples after UHP treatment were all significantly higher than those in the untreated group (*p* < 0.05). The notable content of IDF decreased and that of SDF increased with the prolongation of treatment time. The results are consistent with Gu et al. [30] and Xie et al. [18]. This may be due to the fact that the ultra-high-pressure effect disrupted the structure of dietary fiber. The macromolecular chains in the dietary fiber were broken, and more soluble small molecules were produced. Parts of insoluble cellulose and hemicellulose were converted to IDF [31].

### 3.5. Structural Characterisation of IDFs

#### 3.5.1. Scanning Electron Microscopy (SEM)

**Figure 3:**
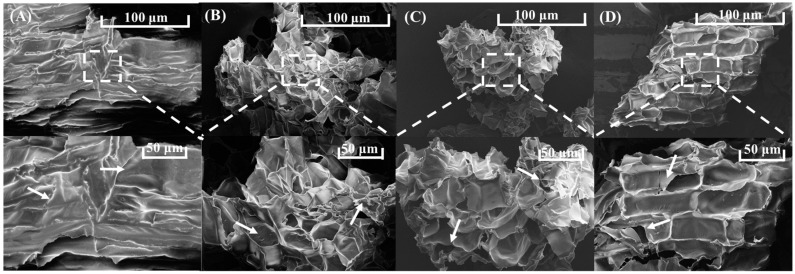
Effect of UHP on the microstructure of insoluble dietary fiber. Scanning electron micrographs of insoluble dietary fiber from coconut chips (**A**), UHP for 20 min (**B**), UHP for 40 min (**C**), and UHP for 60 min (**D**) at magnifications of 200 ×100 μm; 350 ×50 μm, respectively.

The microstructure changes of IDF before and after UHP treatment observed by scanning electron microscopy are shown in Figure 3. The IDF surface in the untreated group (Figure 3A) was relatively smooth, with a dense structure and fewer folds. The surface was adhered by a slight amount of particles, which could be proteins adhered during the IDF extraction process. The hierarchical structure of the IDF was disrupted by the intense ultra-high-pressure action, which caused the surface structure to become rougher than that when untreated. The samples treated for 20 min had more folds on the surface, and the structure became slightly fluffy with the emergence of a porous structure (Figure 3B). After 40 min of treatment, the surface became rough with more folds and pores (Figure 3C). The surface of the IDF treated for 60 min became remarkably rough, with a large number of folds and pores linked together and obvious ruptures. (Figure 3D). The observation of the micro-morphology revealed that the intense physical action generated by UHP could loosen the dense structure of IDF and cause internal damage to IDF [32,33].

#### 3.5.2. Fourier-Infrared (FTIR)

**Figure 4:**
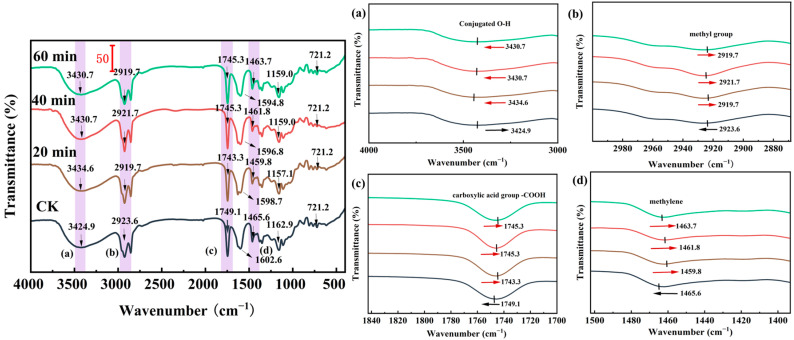
Effect of ultra-high pressure on FTIR spectra of insoluble dietary fiber. CK: untreated group; 20 min, 40 min, and 60 min represent different times of UHP treatment. The four subplots on the right (a), (b), (c), (d) refer to several characteristic absorption peaks with significant variations.

The appearance of a wide and smooth absorption peak of intermediate intensity around 3400 cm^−1^ indicated the contraction vibration of conjugated O-H (Figure 4a) [34]. This was derived primarily from meso-galacturonic acid of pectin and meso-glucuronic acid of hemicellulose, which was the characteristic spectral region of cellulose. These absorption peaks were all shifted compared to those of the untreated group, suggesting that hydrogen bonds in cellulose, hemicellulose, and lignin were disrupted during UHP treatment. The absorption peak near 2923.5 cm^−1^ exhibited a blue shift, and the characteristic peak was mainly caused by the stretching vibration of the methyl group of polysaccharides [35,36]. The following characteristic peaks reflect the basic structure of carbohydrates: the peak at around 1460 cm^−1^ corresponds to the bending vibration peak between the carbon and hydrogen atoms in -CH_2_; the peak at 721.1 cm^−1^ is the in-plane swaying vibration of -CH_2_; and the absorption peak at 1600 cm^−1^ corresponds to the irregular vibration of the carboxyl group (-COOH) in the polysaccharide chain, respectively [37]. The strong peak around 1740 cm^−1^ reflects the C=O structure, and the peaks in the range of 1200–1000 cm^−1^ reflect the presence of C-O [38]. From this it can be deduced that the ester groups were primarily derived from esterified pectin. The intensity of the absorption peaks increased with the extension of UHP treatment time. For example, the intensities of the absorption peaks at 1600 cm^−1^, 1740 cm^−1^, and 2920 cm^−1^ were significantly enhanced compared with those of the untreated peaks at UHP treatment times of 40 min and 60 min. It indicated that the UHP treatment was not able to change the species of the characteristic functional groups. However, it was able to enhance the stretching vibration or bending vibration of the modified characteristic functional groups. This is similar to the findings of Ouyang et al. [33]. Hence, the physicochemical and functional properties of coconut dietary fiber before and after modification would change to a certain extent.

#### 3.5.3. X-ray Diffraction (XRD)

**Figure 5:**
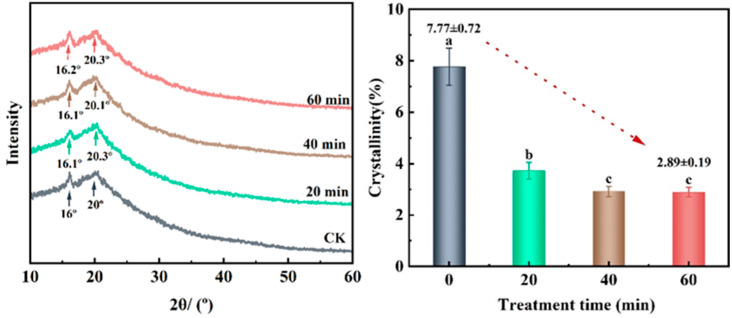
Effect of ultra-high pressure on X-ray diffraction (XRD) of IDF. CK: untreated group; 20 min, 40 min, and 60 min represent different times of UHP treatment. The data for crystallinity are the mean and standard deviation of three measurements. Means with different letters in the graph are significantly different (*p* < 0.05).

There were five crystalline configurations of cellulose in the solid state, which consisted of natural cellulose I, man-made cellulose II, III, IV, and X. As shown in Figure 5, there were characteristic crystalline peaks at about 16° and 20°, corresponding to type I cellulose with a typical double-helix structure [39,40]. The peak patterns were similar among the treatment groups, with intrinsic diffraction peaks appearing at 2θ of 16° and 20° for untreated IDF. The 2θ of IDFs treated for 20 min, 40 min, and 60 min were 16.1°/20.3°, 16.1°/20.1°, and 16.2°/20.3°, respectively. The difference was not significant, which demonstrated that the UHP treatment did not significantly change the crystalline conformation of IDF. The relative crystallinity of the IDFs treated for 20 min, 40 min, and 60 min was found to be 3.72%, 2.92%, and 2.89% after fitting using Jade6.5 (MDI, Livermor, CA, USA). The crystallinity was reduced by 4.45%, 4.85%, and 4.88%, respectively, when compared with 7.77% in the untreated samples. The crystallinity of IDF after UHP treatment all differed from that of the untreated group (*p* < 0.05). The decrease in crystallinity at 40 and 60 min of treatment at 100 MPa was significant compared with that after 20 min of treatment. There was no significant difference between 40 min and 60 min treatment (*p* > 0.05). This was caused by the destruction of the structure of some crystalline regions and the structure of non-crystalline regions in the IDF during the treatment process. The structure changes from ordered to disordered and the molecular polymerization decreased [41]. The structural constituents were rendered soluble or converted into water-soluble components for solubilization. Some crystalline regions were transformed into amorphous regions, and there was degradation of the crystallinity [42]. Similar phenomena have been found in a study by Tan et al. [43]. In conclusion, it can be seen that the difference in the effect of treatment for 40 min and 60 min on IDF its crystallinity was not significant. (*p* < 0.05)

### 3.6. TG and DSC

**Figure 6:**
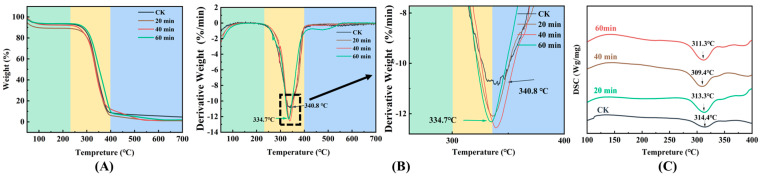
Effect of ultra-high pressure on thermogravimetric (TG) and differential scanning calorimetry (DSC) of IDF. CK: untreated group; 20 min, 40 min, and 60 min represent different times of UHP treatment. TG (**A**,**B**); DSC (**C**).

Thermogravimetric analysis can be used to assess the relationship between sample weight and temperature in addition to assessing the structural properties of IDF. Different chemical components in IDF had different thermal properties, and the temperature of decomposition changed due to the different chemical structures. According to Feiyue Ren et al. [34], the pyrolysis temperature of cellulose ranges from 315 °C to 400 °C, while the pyrolysis temperature of hemicellulose ranges from 210 °C to 350 °C [10]. There were three major stages of thermal weight loss of IDF (Figure 6A,B). The first stage was 50–200 °C, where the weight loss represents mainly the evaporation of water [44]. The second stage was 200–400 °C, which was the thermal decomposition temperature of hemicellulose and cellulose [24]. The sample loses weight at the fastest rate during this phase, which may be due to the breaking of glycosidic and hydrogen bonds in hemicellulose and cellulose. The third stage involved 400–700 °C, where the weight loss rate of the samples slowed down, which may be attributed to the thermal decomposition of the charcoal. The residual amount of IDF was lower than that of the untreated group (2.63%) at 20, 40, and 60 min of UHP treatment (2.61%, 0.98%, and 1.55%, respectively). During DSC determination (Figure 6C), it was found that the IDF showed an absorption peak only after 300 °C. The appearance of this absorption peak was mainly caused by the degradation of cellulose, hemicellulose, and lignin [45]. This result was consistent with the above. The degree of crystallinity decreased, which resulted in the deterioration of the heat resistance of the IDF. A similar observation has been found in the research of Xie et al. [46]. In summary, the thermal stability of IDF was the strongest when affected by UHP treatment for 40 min.

### 3.7. The Monosaccharide Composition of IDF

The composition and proportion of monosaccharides in the IDF samples were determined by high-performance liquid chromatography (HPLC) (Table 3). The main components of hemicellulose were mannose, rhamnose, galacturonic acid, and arabinose. The most significant increase in the content of rhamnose and arabinose was observed after 60 min of treatment compared to that in the untreated group (*p* < 0.05). The most significant increase in mannose content was observed after 40 min of treatment (*p* < 0.05). The content of galacturonic acid decreased in all three treatment groups. Treatment for 60 min resulted in the disappearance of galacturonic acid, which could be attributed to the breakage of glycosidic bonds causing the formation of new monosaccharides under UHP treatment. It was demonstrated that the UHP treatment could change the ratio of the main components in hemicellulose. In addition to this, the content of both glucose as well as glucuronic acid in the IDF after 60 min of treatment was significantly higher (*p* < 0.05) compared to that in the untreated group. This was similar to the results of Liu et al. [47]. It was speculated that it might be due to the fact that IDF was more easily degraded to glucose after UHP modification [33]. The most significant effect was observed under treatment for 60 min, which led to the degradation of hemicellulose. The contents of both arabinose and xylose increased significantly (*p* < 0.05), which indicated the presence of xylan and arabinoxylan. In conclusion, the monosaccharide content and composition of coconut chips were altered, which indicated that the UHP treatment played a role in degrading cellulose and hemicellulose.

## 4. Conclusions

This study was conducted in order to improve the crumbly feeling of coconut chips after chewing. The optimal hardness of coconut chips occurred when the pressure was 100 MPa and pressuring time was 40 min. Hardness was reduced by close to 60%. IDF content dropped by 50%, and the dense structure on the surface of IDF became loose and porous. The stretching or bending vibrations of characteristic functional groups such as hydroxyl, methyl, and carboxyl groups were enhanced. Crystallinity was reduced by 4.92%. The thermal stability deteriorated, and the monosaccharides changed significantly. Hence, this study can contribute to a strong theoretical backup for the improvement of the post-chewing palate of coconut chips after ultra-high-pressure treatment.

## Figures and Tables

**Table 1:** Effect of UHP on main components of coconut chips.

Treatment Group	Moisture Content (%)	Protein (%)	Fat (%)
CK	8.81 ± 1.62 a	7.57 ± 0.88 a	41.37 ± 0.95 b
100 MPa 20 min	4.48 ± 0.65 b	7.10 ± 0.87 a	42.48 ± 0.78 ab
100 MPa 40 min	3.02 ± 0.37 b	7.02 ± 0.69 a	43.58 ± 2.14 a
100 MPa 60 min	3.08 ± 0.73 b	7.8 ± 0.32 a	44.67 ± 0.39 a

All data are averages of three measurements with standard deviation. Means within the same columns with different letters are significantly different (*p* < 0.05). CK: untreated group; 20 min, 40 min, and 60 min represent different times of UHP treatment.

**Table 2:** Effect of UHP on the IDF, SDF, TDF content of coconut chips.

Treatment Group	IDF	SDF	TDF
CK	7.67 ± 0.24 a	1.47 ± 0.30 c	9.23 ± 0.02 b
100 MPa 20 min	6.38 ± 0.12 b	3.14 ± 0.11 b	9.52 ± 0.17 a
100 MPa 40 min	6.13 ± 0.11 b	3.31 ± 0.15 ab	9.44 ± 0.14 ab
100 MPa 60 min	5.59 ± 0.15 c	3.64 ± 0.21 a	9.22 ± 0.06 b

All data are averages of three measurements with standard deviation. Means within the same columns with different letters are significantly different (*p* < 0.05). CK: untreated group; 20 min, 40 min, and 60 min represent different times of UHP treatment. IDF, insoluble dietary fiber; SDF, soluble dietary fiber; TDF, total dietary fiber.

**Table 3:** Effect of UHP on the monosaccharide composition of IDF.

Treatment Group (mol%)	CK	20 min	40 min	60 min
L-Guluronic acid	0.010 ± 0.004 b	0.024 ± 0.001 a	ND	ND
D-Mannuronic acid	0.057 ± 0.031 b	0.103 ± 0.041 c	0.073 ± 0.004 a	0.294 ± 0.002 bc
D-Mannose Man	88.594 ± 0.151 b	88.130 ± 0.540 c	90.320 ± 0.007 a	69.404 ± 0.251 d
L-Rhamnose Rham	0.261 ± 0.003 b	0.163 ± 0.003 b	0.239 ± 0.001 b	0.890 ± 0.141 a
D-Glucuronic acid	0.121 ± 0.006 c	0.12 ± 0.011 c	0.190 ± 0.01 b	11.830 ± 0.053 a
D-Galacturonic acid	0.239 ± 0.006 a	0.144 ± 0.006 c	0.178 ± 0.0153 b	ND
D-glucose	5.002 ± 0.009 c	6.511 ± 0.0205 b	3.925 ± 0.0175 d	7.861 ± 0.115 a
D-galactose	4.482 ± 0.001 a	3.835 ± 0.012 c	3.955 ± 0.065 b	3.221 ± 0.013 d
D-xylulose	ND	0.195 ± 0.002 b	ND	1.096 ± 0.010 a
L-Arabinose	1.234 ± 0.002 b	0.776 ± 0.0065 d	1.120 ± 0.0025 c	3.469 ± 0.010 a
L-Fucose	ND	ND	ND	1.934 ± 0.0410 a

All data are averages of three measurements with standard deviation. Means within the same columns with different letters are significantly different (*p* < 0.05). ND indicates that the substance was not detected.

## Data Availability

The original contributions presented in the study are included in the article, further inquiries can be directed to the corresponding author.

## References

[1] Zhang W., Chan J.X., Lu Y., Liu S.Q. (2022). Pre-treatment of Coconut Kernels by Proteases to Modulate the Flavour of Coconut Oil. Food Biosci..

[2] Zheng Y., Li Y. (2018). Physicochemical and Functional Properties of Coconut (*Cocos nucifera* L.) Cake Dietary Fibres: Effects of Cellulase Hydrolysis, Acid treatment and Particle Size Distribution. Food Chem..

[3] O‘Grady J., O‘Connor E.M., Shanahan F. (2019). Review Article: Dietary Fibre in the era of Microbiome Science. Aliment. Pharm Ther..

[4] Soliman G.A. (2019). Dietary Fiber, Atherosclerosis, and Cardiovascular Disease. Nutrients.

[5] Guan Z.W., Yu E.Z., Feng Q. (2021). Soluble Dietary Fiber, One of the Most Important Nutrients for the Gut Microbiota. Molecules.

[6] Liu T., Lei H., Zhen X., Liu J., Xie W., Tang Q., Gou D., Zhao J. (2024). Advancements in Modifying Insoluble Dietary Fiber: Exploring the Microstructure, Physicochemical Properties, Biological Activity, and Applications in Food Industry—A review. Food Chem..

[7] Gan J., Xie L., Peng G., Xie J., Chen Y., Yu Q. (2021). Systematic Review on Modification Methods of Dietary Fiber. Food Hydrocoll..

[8] Bader Ul Ain H., Saeed F., Khan M.A., Niaz B., Khan S.G., Anjum F.M., Tufail T., Hussain S. (2019). Comparative Study of Chemical Treatments in Combination with Extrusion for the Partial Conversion of Wheat and Sorghum Insoluble Fiber into Soluble. Food Sci. Nutr..

[9] Kanwar P., Yadav R.B., Yadav B.S. (2023). Influence of Chemical Modification Approaches on Physicochemical and Structural Properties of Dietary Fiber from Oat. J. Cereal Sci..

[10] Liu Y., Zhang H., Yi C., Quan K., Lin B. (2021). Chemical Composition, Structure, Physicochemical and Functional Properties of Rice Bran Dietary Fiber Modified by Cellulase Treatment. Food Chem..

[11] Yu G., Bei J., Zhao J., Li Q., Cheng C. (2018). Modification of Carrot (*Daucus carota* Linn. var. *Sativa Hoffm*.) Pomace Insoluble Dietary Fiber with Complex Enzyme Method, Ultrafine Comminution, and High Hydrostatic Pressure. Food Chem..

[12] Zhou D., Liu J., Liu S., Liu X., Tang X., Lv X. (2020). Characterisation of Alkaline and Enzymatic Modified Insoluble Dietary Fibre from Undaria Pinnatifida. Int. J. Food Sci. Technol..

[13] Gao Q., Zhou X.J., Ma R., Lin H., Wu J.L., Peng X., Tanokura M., Xue Y.L. (2021). Hydrogen Peroxide Modification Affects the Structure and Physicochemical Properties of Dietary Fibers from White Turnip (*Brassica rapa* L.). Sci. Rep..

[14] Tian X.Y., Liu J.F., Qiao C.C., Cheng Z., Wu N.N., Tan B. (2024). Functional Properties and Structure of Soluble Dietary Fiber Obtained from Rice Bran with Steam Explosion Treatment. J. Cereal Sci..

[15] Yan J., Li Y., Bai S., Zheng J., Hassan N.A., Lu B., Hu A. (2024). Comparison of Structural, Physicochemical and Functional Properties of Dried Coconut Dietary Fiber by Steam Explosion and Extrusion Modification. Ind. Crops Prod..

[16] Huang Y., Li C., Zheng S., Fu X., Huang Q., Liu G., Chen Q. (2024). Influence of Three Modification Methods on the Structure, Physicochemical, and Functional Properties of Insoluble Dietary Fiber from Rosa Roxburghii Tratt Pomace. Molecules.

[17] Jiang C., Zeng X., Wei X., Liu X., Wang J., Zheng X. (2024). Improvement of the Functional Properties of Insoluble Dietary Fiber from Corn Bran by Ultrasonic-microwave Synergistic Modification. Ultrason. Sonochem..

[18] Xie F., Li M., Lan X., Zhang W., Gong S., Wu J., Wang Z. (2017). Modification of Dietary Fibers From Purple-fleshed potatoes (Heimeiren) with High Hydrostatic Pressure and High Pressure Homogenization Processing: A Comparative Study. Innov. Food Sci. Emerg. Technol..

[19] Zhang Y., Qi J., Zeng W., Huang Y., Yang X. (2020). Properties of Dietary Fiber from Citrus Obtained Through Alkaline Hydrogen Peroxide Treatment and Homogenization Treatment. Food Chem..

[20] Yeager S.E., Batali M.E., Lim L.X., Liang J., Han J., Thompson A.N., Guinard J.-X., Ristenpart W.D. (2022). Roast Level and Brew Temperature Significantly Affect the Color of Brewed Coffee. J. Food Sci..

[21] Serrano S., Rincón F., García-Olmo J. (2013). Cereal protein analysis via Dumas method: Standardization of a micro-method using the EuroVector Elemental Analyser. J. Cereal Sci..

[22] Chutia H., Sharma M., Das M.J., Mahanta C.L. (2024). Properties of Dietary Fibre From Passion Fruit Seed Obtained Through Individual and Combined Alkaline and Ultrasonication Extraction Techniques. Waste Biomass Valoriz..

[23] Begum Y.A., Deka S.C. (2019). Effect of Processing on Structural, Thermal, and Physicochemical Properties of Dietary Fiber of Culinary Banana Bracts. J. Food Process. Preserv..

[24] Zhang W., Zeng G., Pan Y., Chen W., Huang W., Chen H., Li Y. (2017). Properties of Soluble Dietary Fiber-polysaccharide from Papaya Peel Obtained through Alkaline or Ultrasound-Assisted Alkaline Extraction. Carbohydr. Polym..

[25] Du X., Wang L., Huang X., Jing H., Ye X., Gao W., Bai X., Wang H. (2021). Effects of Different Extraction Methods on Structure and Properties of Soluble Dietary Fiber from Defatted Coconut Flour. LWT-Food Sci. Technol..

[26] Xiong M., Zheng S., Bai T., Chen D., Qin W., Zhang Q., Lin D., Liu Y., Liu A., Huang Z. (2022). The Difference among Structure, Physicochemical and Functional Properties of Dietary Fiber Extracted from Triticale and Hull-less Barley. LWT-Food Sci. Technol..

[27] Zhang L., Liao L., Qiao Y., Wang C., Shi D., An K., Hu J. (2020). Effects of Ultrahigh Pressure and Ultrasound Pretreatments on Properties of Strawberry Chips Prepared by Vacuum-freeze Drying. Food Chem..

[28] Zang Z., Wan F., Ma G., Xu Y., Wang T., Wu B., Huang X. (2024). Enhancing Peach Slices Radio Frequency Vacuum Drying by Combining Ultrasound and Ultra-high Pressure as Pretreatments: Effect on Drying Characteristics, Physicochemical Quality, Texture and Sensory Evaluation. Ultrason. Sonochem..

[29] Kang W., Zhang J., Yu N., He L., Chen Y. (2023). Effect of Ultrahigh-pressure Treatment on the Structure and Allergenicity of Peach Allergenic Proteins. Food Chem..

[30] Gu Y., Niu L., Song J., Liu C., Zhang Z., Liu C., Li D., Xiao L. (2022). Effect of Pretreatment and High Hydrostatic Pressure on Soluble Dietary Fiber in Lotus Root Residues. J. Food Qual..

[31] Yang K., Yang Z., Wu W., Gao H., Zhou C., Sun P., Wu C., Xia Q., Chen J. (2020). Physicochemical Properties Improvement and Structural Changes of Bamboo Shoots (*Phyllostachys praecox f. Prevernalis*) Dietary Fiber Modified by Subcritical Water and High Pressure Homogenization: A comparative study. J. Food Sci. Technol..

[32] Zhao S., Pan Z., Azarakhsh N., Ramaswamy H.S., Duan H., Wang C. (2024). Effects of High-Pressure Processing on the Physicochemical and Adsorption Properties, Structural Characteristics, and Dietary Fiber Content of Kelp (*Laminaria japonica*). Curr. Res. Food Sci..

[33] Ouyang H., Guo B., Hu Y., Li L., Jiang Z., Li Q., Ni H., Li Z., Zheng M. (2023). Effect of Ultra-High Pressure Treatment on Structural and Functional Properties of Dietary Fiber from Pomelo Fruitlets. Food Biosci..

[34] Ren F., Feng Y., Zhang H., Wang J. (2021). Effects of Modification Methods on Microstructural and Physicochemical Characteristics of Defatted Rice Bran Dietary Fiber. LWT-Food Sci. Technol..

[35] Honců I., Sluková M., Vaculová K., Sedláčková I., Wiege B., Fehling E. (2016). The Effects of Extrusion on the Content and Properties of Dietary Fibre Components in Various Barley Cultivars. J. Cereal Sci..

[36] Peng Y., Zhang Z., Chen W., Zhao S., Pi Y., Yue X. (2023). Structural Characterization, A-glucosidase Inhibitory Activity and Antioxidant Activity of Neutral Polysaccharide from Apricot (*Armeniaca Sibirica* L. Lam) Kernels. Int. J. Biol. Macromol..

[37] Lettow M., Grabarics M., Mucha E., Thomas D.A., Polewski Ł., Freyse J., Rademann J., Meijer G., von Helden G., Pagel K. (2020). IR Action Spectroscopy of Glycosaminoglycan Oligosaccharides. Anal. Bioanal. Chem..

[38] Liao A.M., Zhang J., Yang Z.L., Huang J.H., Pan L., Hou Y.C., Li X.X., Zhao P.H., Dong Y.Q., Hu Z.Y. (2022). Structural, Physicochemical, and Functional Properties of Wheat Bran Insoluble Dietary Fiber Modified with Probiotic Fermentation. Front. Nutr..

[39] Sun C., Wu X., Chen X., Li X., Zheng Z., Jiang S. (2020). Production and Characterization of Okara Dietary Fiber Produced by Fermentation with Monascus Anka. Food Chem..

[40] Khawas P., Deka S.C. (2016). Isolation and Characterization of Cellulose Nanofibers from Culinary Banana Peel using High-intensity Ultrasonication Combined with Chemical Treatment. Carbohydr. Polym..

[41] Wang D., Liu X., Wang K., Zhao L., Wang Y., Zhang X., Hu Z. (2023). Impact of Non-thermal Modifications on the Physicochemical Properties and Functionality of Litchi Pomace Dietary Fibre. LWT-Food Sci. Technol..

[42] Luo X., Wang Q., Fang D., Zhuang W., Chen C., Jiang W., Zheng Y. (2018). Modification of Insoluble Dietary Fibers from Bamboo Shoot Shell: Structural Characterization and Functional Properties. Int. J. Biol. Macromol..

[43] Tan Y., Li S., Li C., Liu S. (2023). Glucose Adsorption and α-amylase Activity Inhibition Mechanism of Insoluble Dietary Fiber: Comparison of Structural and Microrheological Properties of Three Different Modified Coconut Residue Fibers. Food Chem..

[44] Sung Y.J., Seo Y.B. (2009). Thermogravimetric Study on Stem Biomass of Nicotiana Tabacum. Thermochim. Acta.

[45] Karaman E., Yılmaz E., Tuncel N.B. (2017). Physicochemical, Microstructural and Functional Characterization of Dietary Fibers Extracted from Lemon, Orange and Grapefruit Seeds Press meals. Bioact..

[46] Xie J., Liu S., Dong R., Xie J., Chen Y., Peng G., Liao W., Xue P., Feng L., Yu Q. (2021). Bound Polyphenols from Insoluble Dietary Fiber of Defatted Rice Bran by Solid-State Fermentation with Trichoderma viride: Profile, Activity, and Release Mechanism. J. Agric. Food. Chem..

[47] Liu M., Zhou S., Li Y., Tian J., Zhang C. (2021). Structure, Physicochemical Properties and Effects on Nutrients Digestion of Modified Soluble Dietary Fiber Extracted from Sweet Potato Residue. Food Res. Int..

